# Mutagenesis and redox partners analysis of the P450 fatty acid decarboxylase OleT_JE_

**DOI:** 10.1038/srep44258

**Published:** 2017-03-09

**Authors:** Bo Fang, Huifang Xu, Yi Liu, Fengxia Qi, Wei Zhang, Hui Chen, Cong Wang, Yilin Wang, Wenxia Yang, Shengying Li

**Affiliations:** 1 Shandong Provincial Key Laboratory of Synthetic Biology, CAS Key Laboratory of Biofuels, Qingdao Institute of Bioenergy and Bioprocess Technology, Chinese Academy of Sciences, No. 189 Songling Road, Qingdao 266101, China; 2 University of Chinese Academy of Sciences, Beijing 100049, China

## Abstract

The cytochrome P450 enzyme OleT_JE_ from *Jeotgalicoccus* sp. ATCC 8456 is capable of converting free long-chain fatty acids into α-alkenes via one-step oxidative decarboxylation in presence of H_2_O_2_ as cofactor or using redox partner systems. This enzyme has attracted much attention due to its intriguing but unclear catalytic mechanism and potential application in biofuel production. Here, we investigated the functionality of a select group of residues (Arg245, Cys365, His85, and Ile170) in the active site of OleT_JE_ through extensive mutagenesis analysis. The key roles of these residues for catalytic activity and reaction type selectivity were identified. In addition, a range of heterologous redox partners were found to be able to efficiently support the decarboxylation activity of OleT_JE_. The best combination turned out to be *Se*Fdx-6 (ferredoxin) from *Synechococcus elongatus* PCC 7942 and *Cg*FdR-2 (ferredoxin reductase) from *Corynebacterium glutamicum* ATCC 13032, which gave the highest myristic acid conversion rate of 94.4%. Moreover, Michaelis-Menton kinetic parameters of OleT_JE_ towards myristic acid were determined.

Cytochrome P450 (CYP) enzymes broadly existing in archaea, prokaryotes, and eukaryotes belong to the ubiquitous superfamily composed of diverse functional oxygenases^1^,^2^. These versatile biocatalysts are capable of mediating a great variety of natural and unnatural reactions^2^,^3^,^4^,^5^,^6^. Mechanistically, P450 enzymes can be divided into monooxygenases, peroxidases, and peroxygenases based on their catalytic properties^2^.

P450 monooxygenases utilize O_2_ as oxygen donor and two electrons transferred from NAD(P)H by redox partner protein(s) to the heme iron reactive center, to catalyze the monooxygenation of numerous substrates^3^,^7^. By contrast, P450 peroxygenases, such as P450 OleT_JE_ from *Jeotgalicoccus* sp. ATCC 8456, P450_BSβ_ from *Bacillus subtilis*, and P450_SPα_ from *Sphingomonas paucimobilis*, as members of the CYP152 family^8^,^9^,^10^,^11^,^12^, employ H_2_O_2_ instead of O_2_ as the oxidant as well as the electron donor to catalyze corresponding reactions.

Among these P450 peroxygenases, OleT_JE_ fatty acid decarboxylase has drawn special attentions due to its potential application in biological production of α-alkenes as either biofuels or biomaterials. Catalytically, OleT_JE_ mainly decarboxylates medium-to-long chain (C_12_-C_20_) fatty acids to generate terminal olefins (C_11_-C_19_) using H_2_O_2_ as cofactor. It also catalyzes α- and β-hydroxylation of fatty acids as side reactions^8^,^13^ (Fig. 1). This single-step transformation from free fatty acids to α-alkenes likely represents the most straightforward and efficient route for biosynthesis of aliphatic hydrocarbons, thus being an promising system to be engineered for cost-effective and environmentally sustainable production of fossil fuel alternatives in the future^8^,^14^,^15^.

Since its discovery by Rude *et al*. in 2011^13^, intensive studies have been conducted in order to figure out the substrate specificity^16^,^17^,^18^, the reaction type selectivity (decarboxylation or hydroxylation)^19^, and the unique decarboxylation mechanism of OleT_JE_^8^,^9^,^14^,^15^,^16^. Recently, our laboratory uncovered the H_2_O_2_-independent activity of OleT_JE_. In addition to H_2_O_2_, OleT_JE_ is also able to perform catalysis using the O_2_/redox partner/NADPH system. This result has important mechanistic implication and biotechnological significance^8^. Using alternative redox systems, this P450 enzyme showed different substrate specificity with the preferred substrate being C_12_, C_14_, or C_18_ fatty acids^8^. Makris and coworkers, using transient kinetic isotope effect analysis, recently reported that OleT_JE_ catalysis is initiated by the formation of an iron(IV)-oxo π cation radical (Compound I)^15^. Fatty acid decarboxylation is likely resulted from the subsequent hydrogen abstraction from the C_β_ position of substrate forming a stable Fe^4+^-OH species (compound II), which provides a rationale for the final carbon-carbon scission reaction^15^,^20^. Moreover, the crystal structure of OleT_JE_ in complex with eicosanoic acid (C_20_) strongly suggested an essential role of the active site residues Arg245 and His85 for catalysis and reaction type selectivity, respectively^9^.

These studies have significantly advanced the understanding on the structural basis and catalytic mechanism of OleT_JE_. However, there remain a number of unsolved problems: What are the catalytic residues of OleT_JE_? What are the key amino acids determining whether decarboxylation or hydroxylation would occur? Is it possible to further improve the decarboxylation activity of OleT_JE_ for practical application? Attempting to address these questions, in this work, we performed systematic mutagenesis analysis of four key residues including Arg245, Cys365, His85, and Ile170 to elucidate their functionality. Furthermore, a select group of redox partner proteins were screened in order to identify an optimal decarboxylation system.

## Results and Discussion

### Mutagenesis analysis of OleT_JE_

In the CYP152 family, an arginine residue has been found to be absolutely conserved (Supplementary Fig. S1). In the crystal structures of OleT_JE_, P450_BSβ_, and P450_SPα_^9^,^10^,^21^, the fixation of fatty acid substrate in active site all relies on the salt bridges between the guanidyl group of this arginine (Arg241 in P450_SPα_, Arg242 in P450_BSβ_, and Arg245 in OleT_JE_) and the terminal carboxyl group of fatty acids. Moreover, this arginine located near the heme iron reaction center is thought to be responsible for activating the iron(III)-bound H_2_O_2_ via acid-base catalysis to synthesise Compound I^10^.

Specifically in OleT_JE_, the guanidyl group of Arg245 was observed to be only 2.8 Å away from the carboxyl group of arachidic acid (Fig. 2B). To test if this residue is required for OleT_JE_ activity, a series of single point mutants including R245A, R245Q, R245H, R245L, R245E and R245K were constructed. In the presence of 220 μM H_2_O_2_ and 200 μM myristic acid (C_14_) as substrate, the activities of all Arg245 mutants (2 μM) were compared *in vitro* using the wild type enzyme as positive control. As expected, except for R245K retaining marginal hydroxylation activity perhaps due to the similar chemical property of Lys and Arg, all other mutants were completely inactive (Table 1). These results clearly indicate an essential role of this arginine residue in OleT_JE_ catalysis.

All P450 enzymes including CYP152 peroxygenases unanimously use a cysteine residue to coordinate with the heme iron. By contrast, almost all enzymes (except for chloroperoxidase) in non-P450 peroxygenase superfamily employ a histidine as the iron-coordinating ligand^3^,^7^. In consideration of the key role in O-O scission of the His ligand for peroxygenases^22^, we replaced Cys365 of OleT_JE_ with His to investigate the impact on its peroxygenase activity. As a result, no activity was detected for the OleT_JE_ C365H mutant (Table 1), which indicates this Cys ligand is critical for OleT_JE_ and perhaps other P450 peroxygenases.

Among all CYP152 members that have been biochemically characterized so far, OleT_JE_ is the only one that predominantly catalyzes fatty acid decarboxylation with α- and β-hydroxylation as side reactions. Other CYP152 enzymes including P450_SPα_^12^, P450_BSβ_^23^, and CYP-MP^24^ were identified as fatty acid hydroxylases primarily. P450_SPα_ only hydroxylates fatty acids at α-position^12^. P450_BSβ_ generates α-hydroxy and β-hydroxy fatty acids as major products, and a small amount of α-alkenes^23^. CYP-MP is able to introduce the hydroxyl group at α-, β-, γ-, δ-, and ε-position of C_12_-C_18_ fatty acids, while it only displayed minor decarboxylation activity against myristic acid (C_14_) and palmitic acid (C_16_)^24^. Taken together, it is important to identify the key amino acid residues, structural elements or any other factors responsible for the reaction type selectivity of P450 fatty acid decarboxylase/hydroxylase.

Comparatively, OleT_JE_ and P450_BSβ_ show 41%/60% amino acid sequence identity/similarity. Their overall structures and active site compositions are highly similar to each other (Fig. 2). The only two obviously different residues in their active sites are His85 (Gln85 in P450_BSβ_) and Ile170 (Val170 in P450_BSβ_), which reside at the two sides of the carboxyl group of substrate with a distance of 5.1 Å and 3.4 Å, respectively, in the crystal structure of OleT_JE_ (Fig. 2). Thus, we hypothesize that these two amino acids might be related to the decarboxylation/hydroxylation selectivity.

To test this hypothesis, saturation mutagenesis of His85 and Ile170 in OleT_JE_ was performed, and the *in vitro* activities of H85X and I170X variants towards myristic acid were evaluated (Fig. 3). As results, 11 out of 19 H85X variants were completely dead mutants. The rest 8 mutants unanimously lost their decarboxylation activities, while retaining varying hydroxylation activities (Fig. 3A). Notably, the two substitutions with an amide side chain (H85Q and H85N) retained most hydroxylation activity for unknown reasons.

Previously, Rude *et al*. proposed the importance of His85 for the decarboxylation activity of OleT_JE_ based on the result that the Q85H mutation of P450_BSβ_ enhanced its decarboxylation activity towards palmitic acid for about 50%^13^, which led to an increase in the ratio of decarcarboxylation to hydroxylation from 0.19 in wild type P450_BSβ_ to 0.30 in the P450_BSβ_ Q85H mutant. In this work, we confirmed the necessity of His85 for the decarboxylation activity of OleT_JE_. This is well consistent with the mechanism proposed by Belcher *et al*., in which His85 could act as a proton donor to compound I. This proton donation resulting in the protonated compound II was thought to be required for generation of the carboxylate radical for homolytic scission of the substrate C-C_α_ bond, thereby forming the terminal alkene. In the absence of proton donor, hydroxylation would be the only reaction^9^,^20^.

Similarly, none of the I170X mutants showed any decarboxylation activity against myristic acid. However, 11 out of 19 variants were able to catalyze the α- and/or β-hydroxylation to different extents (Fig. 3B). Thus, the mutagenesis analysis of His85 and Ile170 clearly indicates that these two residues adjacent to the carboxyl end of substrate are key factors for fatty acid decarboxylation.

Together with Arg245, these three amino acids are likely involved in the exact substrate positioning required for decarboxylation, explaining why conserved substitutions (e.g. I170V or I170L) also abolished the decarboxylation activity. In this regard, it might be highly challenging to rationally design a better version of OleT_JE_ that is more selective for decarboxylation than hydroxylation without compromising the total activity, at least based on the current knowledge on the structure-function relationship of P450 fatty acid decarboxylases. A recent study of site-directed mutagenesis of OleT_JE_ at selected sites lining the substrate binding pocket also proved difficulty in improving OleT_JE_ activities towards structurally different aromatic carboxylic acid substrates. Only meager improvements (less than 1-fold) were observed in the few positively responded mutants (F79L and F294A)^17^. To overcome these rational design challenges, random gene mutagenesis or DNA shuffling coupled to high-throughput screening could be a more feasible strategy.

### *In vitro* decarboxylation activity of OleT_JE_ supported by different redox partners

**Figure 1:**
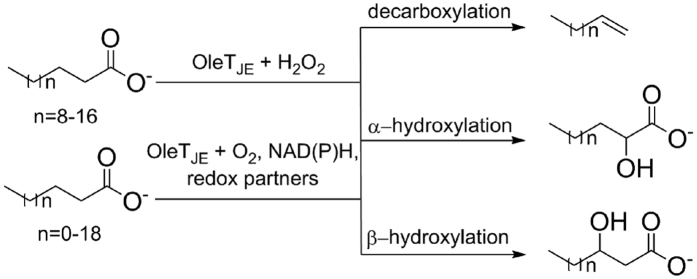
Decarboxylation and hydroxylation of fatty acids catalyzed by OleT_JE_.

**Figure 2:**
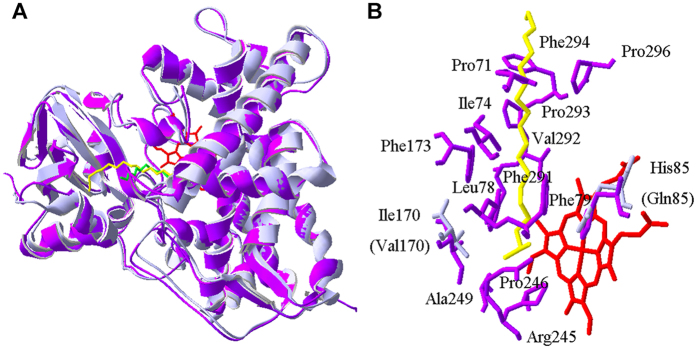
Comparison of overall structures and substrate binding pockets of OleT_JE_ and P450_BSβ_. (**A**) Structural superimposition of OleT_JE_ (in purple, PDB ID code 4L40) and P450_BSβ_ (in grey, PDB ID code 1IZO); (**B**) Comparison of substrate binding pockets between OleT_JE_ and P450_BSβ_. Red: heme iron; yellow: eicosanoic acid for OleT_JE_; green: palmitic acid for P450_BSβ_; purple: major active site residues in OleT_JE_; grey: major different amino acids in P450_BSβ_.

**Figure 3:**
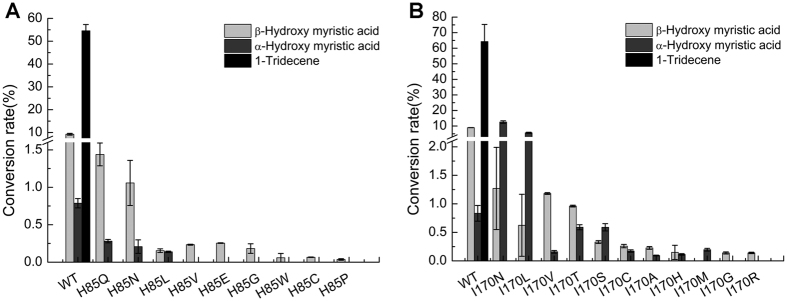
Decarboxylation and hydroxylation reactions catalyzed by OleT_JE_ and its mutants H85X (**A**) and I170X (**B**). Reaction conditions: wild type or mutant enzymes (2 μM), H_2_O_2_ (220 μM), and myristic acid (200 μM) in 200 μl desalting buffer were incubated at 30 °C for 16 h. All experiments were performed in duplicate.

When OleT_JE_ was first identified to be a P450 fatty acid decarboxylase with potential application in the field of biofuels, it was thought to be an obligate peroxygenase as P450_SPα_ and P450_BSβ_. However, our laboratory recently revealed the H_2_O_2_-independent activity of OleT_JE_ (i.e. the activity depending on O_2_/redox partner(s)/NAD(P)H). This discovery has initiated the development of different olefin producing systems based on OleT_JE_ and alternative redox partner protein(s). For instance, we have shown that the flavodoxin/flavodoxin reductase from *E. coli* and the RhFRED reductase from *Rhodococcus* sp. NCIMB 9784 are capable of supporting the OleT_JE_ activity both *in vitro* and *in vivo*^8^. Dennig *et al*. employed putidaredoxin and putidaredoxin reductase from *Pseudomonas putida* to achieve the decarboxylation of short-chain fatty acids (C_4_-C_9_) into corresponding α-alkenes *in vitro*^14^ (Fig. 1). More importantly, by taking advantage of heterologous P450 redox partners, the engineered *E. coli*^8^ and *Saccharomyces cerevisiae*^25^ cells with OleT_JE_ expression were able to produce 97.6 mg/L and 3.7 mg/L total α-alkenes, respectively.

**Figure 4:**
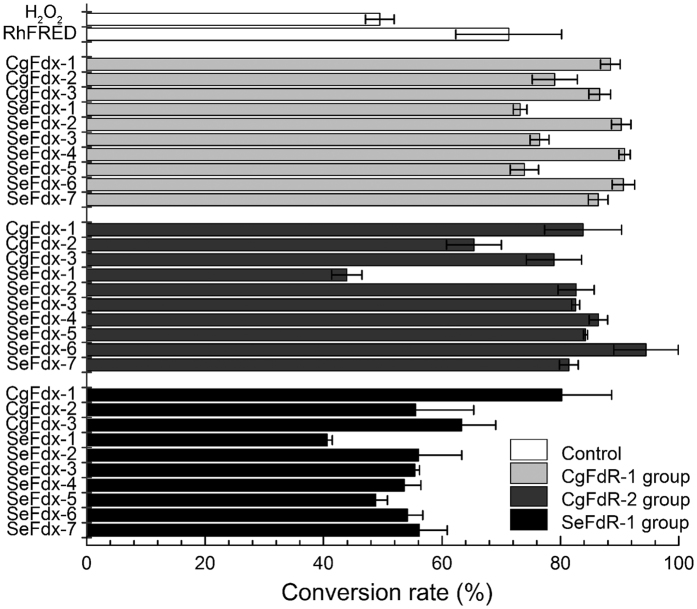
Myristic acid decarboxylation reactions catalyzed by OleT_JE_ when partnered with different redox systems. Reaction conditions: OleT_JE_ (1 μM), NADPH (5 mM), myristic acid (400 μM), ferredoxin (20 μM), and ferredoxin reductase (20 μM) in 100 μl desalting buffer were incubated at 30 °C for 16 h. All experiments were performed in duplicate.

To identify the H_2_O_2_-independent activity of OleT_JE_ with different redox partners, we *in vitro* screened a series of ferredoxins (Fdx) and ferredoxin reductases (FdR) derived from the cyanobacterial strain *Synechococcus elongatus* PCC 7942 and the Gram-positive bacterium *Corynebacterium glutamicum* ATCC 13032 (Supplementary DNA sequences of redox partners). Specifically, three FdRs (*Se*FdR-1 from *S. elongates*, and *Cg*FdR-1 and *Cg*FdR-2 from *C. glutamicum*) were individually coupled with ten Fdxs (*Se*Fdx-1–7 from *S. elongatus* and *Cg*Fdx-1–3 from *C. glutamicum*), and 30 different combinations of redox partner proteins were mixed with OleT_JE_, myristic acid, and NADPH, respectively. The supportive activities of all redox partner combinations were compared to that of RhFRED and H_2_O_2_ (Fig. 4). Interestingly, all tested hybrid redox systems were able to support the *in vitro* decarboxylation activity of OleT_JE_ to some extent, indicating the low selectivity of redox partners by this P450 fatty acid decarboxylase. The best combination turned out to be *Cg*FdR-2 and *Se*Fdx-6, which gave the highest conversion rate of 94.4% (Fig. 4). Using these two optimal redox partner proteins to mediate the electron transfer from NADPH, the steady-state kinetic parameters of OleT_JE_ towards myristic acid were determined to be *K*_*m*_ = 5.0 ± 2.4 μM, *k*_*cat*_ = 2.2 ± 0.2 min^−1^, and *k*_*cat*_/*K*_*m*_ = 0.4 μM^−1^ min^−1^ (Supplementary Fig. S2A). Comparatively, the values of *K*_*m*_ and *k*_*cat*_ were 24.2 ± 8.7 μM and 71.0 ± 8.4 min^−1^ (Supplementary Fig. S2B), respectively, when H_2_O_2_ was employed as the sole oxygen and electron donor. The *k*_*cat*_/*K*_*m*_ value of 2.9 μM^−1^ min^−1^ greater than 0.4 μM^−1^ min^−1^ seemed inconsistent with the qualitative results that the *Cg*FdR-2/*Se*Fdx-6/NADPH redox system showed higher fatty acid to α-alkene conversion rate (94.4%) than that supported by H_2_O_2_ (49.5%). We reason this contradiction might be due to inactivation of OleT_JE_ by H_2_O_2_ during prolonged incubation. Taken together, these results demonstrated efficient monooxygenase-like property of OleT_JE_ to use the O_2_/redox partner(s)/NAD(P)H system, which is critical for the future investigation of the unique mechanism and better application of this enzyme.

## Conclusion

We have systematically investigated the functions of three active site residues of OleT_JE_ including Arg245, His85, and Ile170 by site-directed mutagenesis. It was found that they are all required for the decarboxylation activity of OleT_JE_, presumably by forming a salt-bridge with the substrate carboxyl group (Arg245), by acting as a proton donor (His85), and by precisely coordinating substrate positioning in the active site (Ile170). We also studied the H_2_O_2_-independent activity of OleT_JE_ and revealed a series of heterologous redox partners capable of supporting its decarboxylation activity efficiently *in vitro*. These results not only further our understanding on the unique decarboxylative mechanism of OleT_JE_, but also serve as a guide for further bioengineering of this P450 system and the future industrial application.

## Methods

### Reagents

The recombinant plasmid pET28b-*oleT*_*JE*_ for overexpression of the P450 enzyme OleT_JE_ was constructed by our laboratory previously^8^. Fatty acids (myristic acid and heptadecanoic acid), 1-tridecene authentic standards, and derivatizing reagent BSTFA-TMCS were purchased from TCI (Shanghai, China). Antibiotics and isopropyl β-D-1-thiogalactopyranoside (IPTG) were obtained from Solarbio (Beijing, China). All restricted enzymes were purchased from Thermo Scientific (Shanghai, China). PrimeSTAR GXL DNA polymerase was obtained from Takara (Otsu, Japan). Kits used for DNA manipulation were bought from OMEGA Bio-Tek (Jinan, China) or Promega (Madison, WI, USA). Ni-NTA resin from Qiagen (Valencia, CA, USA), Millipore Amicon Ultra centrifugal fliters (Billerica, MA, USA) and PD-10 desalting columns purchased from GE Healthcare (Piscataway, NJ, USA) were used for protein purification.

### Strains, plasmids and media

*Escherichia coli* DH5α cells were used for plasmid transformation and mutant screening. *Escherichia coli* BL21(DE3) was used for protein overexpression. The plasmid pET28b was used for gene cloning. *E. coli* cells were grown in Terrific Broth medium composed of 1.2% tryptone, 0.5% glycerol, 2.4% yeast extract, 0.23% KH_2_PO_4_ and 1.25% K_2_HPO_4_, supplemented with the selective antibiotic (50 μg/mL kanamycin), thiamine (1 mM) and rare salt solution for protein expression^26^.

### Molecular cloning

With pET28b-*oleT*_*JE*_ as template, the *oleT*_*JE*_ mutants were constructed using the Quikchange mutagenesis method and cloned into pET28b vector. Mutagenesis primers are listed in Supplementary Table S1.

The coding DNA sequences of ferredoxin reductase *Cg*FdR-1 and *Cg*FdR-2 were amplified from *Corynebacterium glutamicum* ATCC 13032. The coding DNA sequences of seven ferredoxins *Se*Fdx-1, *Se*Fdx-2, *Se*Fdx-3, *Se*Fdx-4, *Se*Fdx-5, *Se*Fdx-6 and *Se*Fdx-7 from *Synechococcus elongatus* PCC 7942 were codon-optimized by Genewiz (Suzhou, China) and cloned into pET28b. The other three ferredoxin genes encoding *Cg*Fdx-1, *Cg*Fdx-2 and *Cg*Fdx-3 were PCR amplified from *Corynebacterium glutamicum* ATCC 13032. The primers are listed in Supplementary Table S2. The *NdeI* and *XhoI* restriction sites were used for cloning into the *NdeI/XhoI* pre-treated pET28b to obtain corresponding expression vectors.

### Overexpression and purification of proteins

The recombinant expression plasmids were transformed into *E. coli* BL21(DE3). After cultivation in LB medium containing 50 μg/mL kanamycin at 37 °C, 220 rpm overnight, 1% volume of the seed culture was used to inoculate 1 L Teffific Broth medium containing 50 μg/mL kanamycin, 1 mM thiamine and rare salt solution. When the OD_600_ reached 0.6–0.8, IPTG was added to a final concentration of 0.2 mM for induction, followed by shaking incubation at 18 °C for 20 h.

After harvesting by centrifugation at 4 °C, 6000 rpm for 10 min, cells were stored at −80 °C for 30 min. Then cells were thawed and re-suspended in 40 mL lysis buffer (50 mM NaH_2_PO_4_, 300 mM NaCl, 10% glycerol and 10 mM imidazole, pH 8.0) and sonicated on a JY92-IIDN ultra-sonicator for 30 min with a 5 s on and 5 s off pulse. The whole cell lysates were centrifuged at 12,000 rpm for 30 min at 4 °C. The clarified cell lysates were collected and incubated with 1 mL Ni-NTA resin under gentle shaking at 4 °C for 1–2 h. The mixture was then loaded onto an empty column and washed with approximately 100 mL wash buffer (50 mM NaH_2_PO_4_, 300 mM NaCl, 10% glycerol and 20 mM imidazole, pH 8.0) until no proteins were detected in the flow-through. The bound protein was eluted by 10 mL elution buffer (50 mM NaH_2_PO_4_, 300 mM NaCl, 10% glycerol and 250 mM imidazole, pH 8.0) and then concentrated using a Millipore Ultra-filter (30 K). The concentrated protein solution was loaded onto a PD-10 column, which was used for removal of imidazole and buffer exchange into desalting buffer (50 mM NaH_2_PO_4_ and 10% glycerol, pH 7.4). The eluent was flash-frozen by liquid nitrogen and stored at −80 °C for later use.

### Determination of enzyme concentration

The ferric-CO complex of P450 enzyme was prepared by slow bubbling of carbon monoxide gas into a solution of purified ferric P450 for 1 min. Then its UV-visible absorption spectrum from 300 nm to 600 nm was recorded on a UV-visible spectrophotometer DU800 (Beckman Coulter, Fullerton, CA, USA). Following reduction by sodium dithionite, the corresponding spectrum of reduced ferrous-CO adducts was recorded. The functional concentration of P450 was calculated from the CO-bound reduced differential spectrum using a molar extinction coefficient (ε_450–490_) of 91 mM^−1^.cm^−1^ 27.

The concentration of purified ferredoxin and ferredoxin reductase was determined by monitoring the absorbance at 325 nm and 454 nm, respectively, and using their corresponding molar extinction coefficient 15.4 mM^−1^.cm^−1^ (ε_325_) and 11.3 mM^−1^.cm^−1^ (ε_454_)^28^.

### *In vitro* enzymatic assays of OleT_JE_ variants with H_2_O_2_ as sole cofactor

Fatty acid decarboxylation and hydroxylation assays of purified OleT_JE_ variants were performed in 200 μL reaction mixture containing 2 μM OleT_JE_ or OleT_JE_ mutants, 220 μM H_2_O_2_, and 200 μM myristic acid as substrate in desalting buffer. The reactions were carried out at 30 °C for 16 h and then quenched with 20 μL of 10 M HCl. After adding 500 μM heptadecanoic acid as the internal standard, 150 μL of ethyl acetate was used for extraction. The mixture was separated into aqueous and organic phase by centrifugation at 14,000 rpm for 10 min. 70 μL of the organic phase was derivatized with an equal volume of *N,O*-bis (trimethylsilyl) trifluoroacetamide with 1% trimethylchlorosilane (BSTFA-TMCS) at 70 °C for 15 min. Samples were then analyzed using gas chromatography-mass spectroscopy (GC-MS).

### *In vitro* enzymatic assays of OleT_JE_ with the O_2_/NAD(P)H/redox partners system

Fatty acids decarboxylation of purified OleT_JE_ supported by various heterologous redox partners were performed in 100 μL desalting buffer containing 1 μM OleT_JE_, 5 mM NAD(P)H, 400 μM myristic acid substrate, 20 μM ferredoxin and 20 μM ferredoxin reductase. The reactions were carried out at 30 °C for 16 h and then quenched with 10 μL of 10 M HCl. After adding 500 μM heptadecanoic acid as the internal standard, 150 μL of ethyl acetate was used for extraction. Samples were then analyzed using GC-MS.

### Steady-state kinetics

For the reaction system using H_2_O_2_ as the sole oxygen and electron donor, the standard reactions containing 50 nM of OleT_JE_, 20–100 μM myristic acid in 1.2 mL of desalting buffer (50 mM NaH_2_PO_4_ and 10% glycerol, pH 7.4) were pre-incubated at 30 °C for 5 min. The reactions were then initiated by adding 220 μM H_2_O_2_. For the O_2_/*Cg*FdR-2/*Se*Fdx-6/NADPH system, the standard reactions containing 100 nM of OleT_JE_, 10–50 μM myristic acid, 2 μM *Cg*FdR-2 from *C. glutamicum* ATCC 13032 and 2 μM *Se*Fdx-6 from *Synechococcus elongatus* PCC 7942 in 1.2 mL of desalting buffer (50 mM NaH_2_PO_4_ and 10% glycerol, pH 7.4) were pre-incubated at 30 °C for 5 min. The reactions were then initiated by adding 500 μM NADPH. For all reactions, 200 μL aliquots were taken at four different time points to be stopped by adding 1/10 volume of 10 M HCl. Sample extraction was performed as above with 500 μM heptadecanoic acid as the internal standard. For *K*_*m*_ and *V*_*max*_ determinations, the initial velocity of product formation was monitored by GC-MS. Kinetic data from duplicated experiments were fit into Michaelis-Menten equation using Origin 8.0 program.

### Analytical method

Qualitative and quantitative analysis of the products were performed by GC-MS^29^. To detect α-alkenes, an Agilent 7890A gas chromatography equipped with HP-INNOWAX (Agilent Technologies, Inc., cross-linked polyethylene glycerol, Santa Clara, CA, USA; 0.25 µm film thickness, 30 m by 0.25 mm) column was adopted. The column heating program was as follows: the initial temperature of oven was set to 40 °C for 4 min, then increased at a rate of 10 °C/min to 250 °C, and hold for 15 min. The α- and β-hydroxy fatty acids detection was carried out using the Agilent J&W DB-5 MS column (0.25 μm film thickness, 30 m by 0.25 mm). Furthermore, an Agilent 5975C MSD quadrupole mass spectrometer with a scan range from 50 to 500 m/z under electron ionization condition (70 eV) was coupled to the GC. The oven temperature was 50 °C initially and ramped up to 300 °C at the above mentioned rate, then 300 °C for 5 min. Quantification was performed using the corresponding authentic standard compounds and heptadecanoic acid as the internal standard.

## Additional Information

**How to cite this article**: Fang, B. *et al*. Mutagenesis and redox partners analysis of the P450 fatty acid decarboxylase OleT_JE_. *Sci. Rep.* **7**, 44258; doi: 10.1038/srep44258 (2017).

**Publisher's note:** Springer Nature remains neutral with regard to jurisdictional claims in published maps and institutional affiliations.

## Supplementary Material

Supplementary Information

## Figures and Tables

**Table 1:** The catalytic activities of wild type and mutant OleT_JE_.

Enzyme	Conversion rate (%)	Product distribution (%)		
		α-hydroxy myristic acid	β-hydroxy myristic acid	1-tridecene
OleT_JE_	76.9 ± 9.6	1.4 ± 0.1	12.5 ± 0.1	86.2 ± 2.0
R245Q	0	n.d.	n.d.	n.d.
R245H	0	n.d.	n.d.	n.d.
R245E	0	n.d.	n.d.	n.d.
R245K	0.8 ± 0.1	13.8 ± 0.1	86.3 ± 0.1	n.d.
R245A	0	n.d.	n.d.	n.d.
R245L	0	n.d.	n.d.	n.d.
C365H	0	n.d.	n.d.	n.d.

Reaction conditions: wild type and mutant OleT_JE_ (2 μM), H_2_O_2_ (220 μM), myristic acid (200 μM) in 200 μl desalting buffer were incubated at 30 °C for 16 h. All experiments were performed in duplicate. n.d.: not detected.
