# Supplementary material for: Mutagenesis and redox partners analysis of the P450 fatty acid decarboxylase OleTJE
Source: Sci Rep. 2017 Mar 9;7:44258. doi: 10.1038/srep44258 (PMC5343568; doi:10.1038/srep44258)
Supplement: Supplementary Information [file srep44258-s1.pdf]

Supplementary Information

Mutagenesis and redox partners analysis of the P450 fatty acid decarboxylase OleT$_{JE}$

Bo Fang[1,2‡], Huifang Xu[1‡], Yi Liu[1], Fengxia Qi[1], Wei Zhang[1], Hui Chen[1], Cong Wang[1], Yilin Wang[1,2], Wenxia Yang[1], and Shengying Li[1]*

[1]Shandong Provincial Key Laboratory of Synthetic Biology, CAS Key Laboratory of Biofuels, Qingdao Institute of Bioenergy and Bioprocess Technology, Chinese Academy of Sciences, No. 189 Songling Road, Qingdao 266101,China.

[2]University of Chinese Academy of Sciences, Beijing 100049, China

*Corresponding author Shengying Li

[‡]These authors contribute equally to this work.

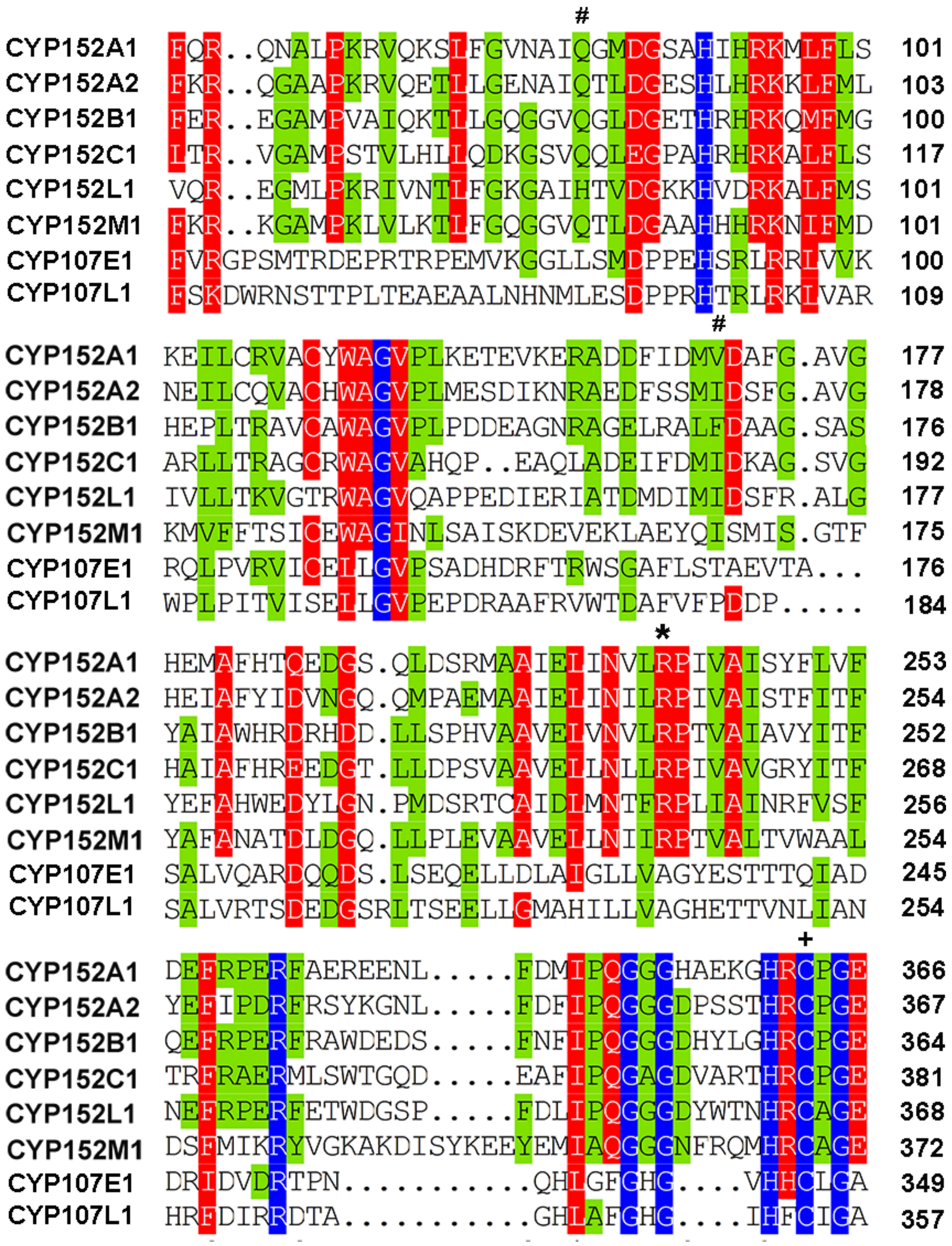

Supplementary Figure S1 Protein sequence alignment of the select group of CYP152 family members along with two P450 monooxygenase sequences from the CYP107 family. CYP152A1: P450$_{BS\beta}$ from *Bacillus subtilis*; CYP152A2: P450$_{CLA}$ from *Clostridium acetobutylicum*; CYP152B1: P450$_{SP\alpha}$ from *Sphingomonas paucimobilis*; CYP152C1: from *Rhodobacter sphaeroides*; CYP152L1: OleT$_{JE}$ from *Jeotgalicoccus* sp. ATCC 8456; CYP152M1: from *Enterococcus faecium*; CYP107E1: monooxygenase P450 MycG from *Micromonospora griseorubida*; CYP107L1: monooxygenase P450 PikC from *Streptomyces venezuelae*; #: Residues important for product distribution; *: the key catalytic residue; +: the absolutely conserved Cys in P450s.

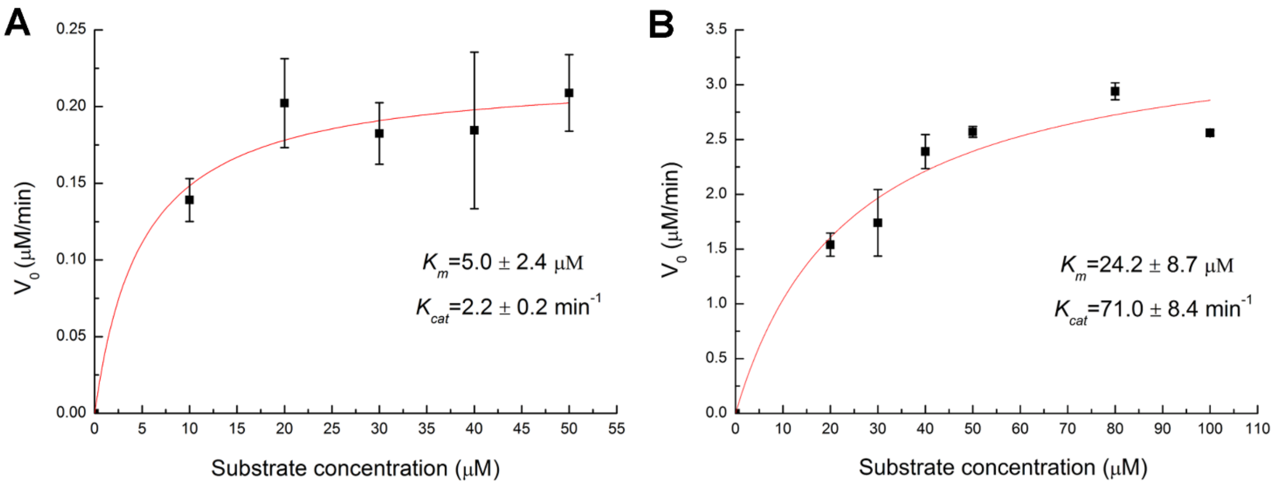

Supplementary Figure S2 The kinetic curves of $OleT_{JE}$ toward myristic acid using (A) the O_2/NADPH/*Cg*FdR-2/*Se*Fdx-6 system, and (B) H_2O_2 as the sole oxygen and electron donor .

Supplementary Table S1 Primers used for site-directed mutagenesis (The bold nucleotides denote the mutated codons.)

Primer names	Sequence 5→3
R245A-F	*GACTTAATGAACACATTC**GCC**CCATTAATCGCAATCAAC*
R245A-R	*GTTGATTGCGATTAATGG**GGC**GAATGTGTTCATTAAGTC*
R245L-F	*GACTTAATGAACACATTC**CTC**CCATTAATCGCAATCAAC*
R245L-R	*GTTGATTGCGATTAATGG**GAG**GAATGTGTTCATTAAGTC*
R245K-F	*GACTTAATGAACACATTC**AAG**CCATTAATCGCAATCAAC*
R245K-R	*GTTGATTGCGATTAATGG**CTT**GAATGTGTTCATTAAGTC*
R245H-F	*GACTTAATGAACACATTC**CAC**CCATTAATCGCAATCAAC*
R245H-R	*GTTGATTGCGATTAATGG**GTG**GAATGTGTTCATTAAGTC*
R245E-F	*GACTTAATGAACACATTC**GAG**CCATTAATCGCAATCAAC*
R245E-R	*GTTGATTGCGATTAATGG**CTC**GAATGTGTTCATTAAGTC*
R245Q-F	*GACTTAATGAACACATTC**CAG**CCATTAATCGCAATCAAC*
R245Q-R	*GTTGATTGCGATTAATGG**CTG**GAATGTGTTCATTAAGTC*
C365H-F	*GGACAAATCACCGT**CAT**GCAGGTGAATGGATC*
C365H-R	*GATCCATTCACCTGC**ATG**ACGGTGATTTGTCC*
I170X-F[a]	*CAGACATGGACATCATG**NNN**GATTCATTTAGAGCAC*
I170X-R[a]	*GTGCTCTAAATGAATC**NNN**CATGATGTCCATGTCTG*
I170R-F[b]	*CAGACATGGACATCATG**CGT**GATTCATTTAGAGCAC*
I170R-R[b]	*GTGCTCTAAATGAATC**ACG**CATGATGTCCATGTCTG*
I170D-F[b]	*CAGACATGGACATCATG**GAC**GATTCATTTAGAGCAC*
I170D-R[b]	*GTGCTCTAAATGAATC**GTC**CATGATGTCCATGTCTG*
I170S-F[b]	*CAGACATGGACATCATG**AGC**GATTCATTTAGAGCAC*
I170S-R[b]	*GTGCTCTAAATGAATC**GCT**CATGATGTCCATGTCTG*
H85X-F[a]	*GTAAAGGTGCAATC**NNN**ACGGTAGATGGT*
H85X-R[a]	*TACCATCTACCGT**NNN**GATTGCACCTTTAC*
H85Y-F[b]	*GTAAAGGTGCAATC**TAT**ACGGTAGATGGT*
H85Y-R[b]	*TACCATCTACCGT**ATA**GATTGCACCTTTAC*
H85F-F[b]	*GTAAAGGTGCAATC**TTC**ACGGTAGATGGT*
H85F-R[b]	*TACCATCTACCGT**GAA**GATTGCACCTTTAC*
H85P-F[b]	*GTAAAGGTGCAATC**CCT**ACGGTAGATGGT*
H85P-R[b]	*TACCATCTACCGT**AGG**GATTGCACCTTTAC*
H85W-F[b]	*GTAAAGGTGCAATC**TGG**ACGGTAGATGGT*
H85W-R[b]	*TACCATCTACCGT**CCA**GATTGCACCTTTAC*
H85M-F[b]	*GTAAAGGTGCAATC**ATG**ACGGTAGATGGT*
H85M-R[b]	*TACCATCTACCGT**CAT**GATTGCACCTTTAC*

[a]for saturation mutagenesis

[b]for the mutants that were not obtained from screening

Supplementary Table S2 Primers used for cloning (The underlined bases denote the restriction site.)

Primer names	Sequence 5'→3'
*Cg*Fdx-3-NdeI	*GCAATATTCATATGATGACCTACACAATCGCC*
*Cg*Fdx-3-XhoI	*GAATTCTCGAGCTAGTTCTGGTTCTGTGG*
*Cg*Fdx-2-NdeI	*GGCCGGCCATATGATGTCTACTATTCATTTC*
*Cg*Fdx-2-XhoI	*GAATTCTCGAGTCACACTTGCGTTTCTGG*
*Cg*Fdx-1-NdeI	*GGCCGGCCATATGATGACATACACAATCGCA*
*Cg*Fdx-1-XhoI	*GAATTCTCGAGTTATGCCTGAGGCGGCAG*
*Cg*FdR-2-NdeI	*GAATTATTCATATGATGTCTCGCCCTTTGCGT*
*Cg*FdR-2-XhoI	*GGCTTCTCGAGTTAGATGTCGTATTCTGG*
*Cg*FdR-1-NdeI	*GAATTATTCATATGATGACAACTCCCCTGCGC*
*Cg*FdR-1-XhoI	*GGCTTCTCGAGTTAGACAATTGCTGGAGC*

Supplementary DNA sequences of redox partners

1. Ferredoxins

(1) Codon-optimized *Se*Fdx-1 (GenBank Accession No. SYNPCC7942_2581)

ATGAGCGATACCTATACCGTTCGCATTCGCGATCGCCGCACCGATGA
AGAATTTACCGTGCAGGTGCCGCCGGATCGCTATATTCTGCAGACCGCCG
AAGAACAGGGCTATGAACTGCCGTTTAGCTGCCGTAATGGCGCCTGCAC
CGCATGTGCCGTTCGTGTGCTGGGTGGTGCCATTGAACAGACCGAAGCA
ATGGGTCTGAGTGCACCGCTGCGTCAGCGTGGTTATGCCCTGCTGTGTG
TGAGCTATCCGCGCAGTGATGTGATTGTGGAGACCCAGGACGAAGATGA
GGTGTACATGCTGCAGTTCGGTCGCTACTTTGGTCAGGGCAAGGTGAGC
TTTGGTCTGCCGCTGGATGAAGAATAA

(2) Codon-optimized *Se*Fdx-2 (GenBank Accession No. SYNPCC7942_1749)

ATGACCACCCCGGATCGTAGCGGTCTGGAGCCGGAACTGGGCGGTA
GTCTGCGTCATGGTCAGGCACGCAGTGGTCTGGAACCGGAGCTGGGTG
GCGAACTGCGTCAGAAACTGGTGTGGGTGGATGAGGTGACCTGCATTGG
CTGCCGTTATTGCAGCCATGTGGCCACCAACACCTTCTACATCGAGCCGG
ATTATGGCCGTAGCCGCGTGGTGCGCCAGAATGGTGATCCGGAAGAACT
GGTGCAGGAAGCCATTGATACCTGCCCGGTGGATTGCATCCATTGGGTG
AATCCGAGCGAACTGCGCCAGCTGGAAGCCGAACGCCGCAATCAGGTG
ATTATGCCGCTGGGTTTTCCGCAGGAACGCAGCAAACAGCGCCGCCGTA
CCTAA

(3) Codon-optimized *Se*Fdx-3 (GenBank Accession No. SYNPCC7942_1499)

ATGGCCACCTATAAAGTGACCCTGGTGAACGCCGCCGAAGGCCTGA
ATACCACCATTGATGTGGCCGACGACACCTATATTCTGGATGCCGCCGAA
GAACAGGGCATTGATCTGCCGTATAGCTGCCGCGCAGGTGCCTGTAGTA
CCTGCGCAGGTAAAGTGGTGAGCGGTACCGTGGATCAGAGCGATCAGAG
CTTCCTGGATGATGATCAGATTGCCGCCGGTTTTGTGCTGACCTGCGTGG
CCTATCCGACCAGCGATGTGACCATCGAAACCCACAAAGAAGAAGATCTG
TATTAA

(4) Codon-optimized *Se*Fdx-4 (GenBank Accession No. SYNPCC7942_0898)

ATGACCCTGGCCGAAACCCTGAGCGTTCGTGTTCGCAGCCTGGGCC
TGGACCAGATTGACCGTCATCTGTTTCTGTGCGCCGATCAGACCAAACC
GCTGTGCTGCGATCGCGATCGCAGCCTGGAAAGCTGGGAGTATCTGAAA
CGCCGCCTGCGTGAACTGGATCTGGATCGCCCGGATACCGGTAAACCGC
TGGTGTTTCGCACCAAAGCCAATTGCCTGCGCGTGTGCCAGGAAGGTCC
GATTCTGCTGGTGTATCCGGAAGGCATTTGGTATGGTCGCGTGACCCCG
GAAGCCATTGAACGCATTCTGCAGGAACACCTGCTGGGTGGTCAGCCGG
TGCAGGAACTGATTCTGCATCAGCATGCCCTGCCGGCAGTGGATCCGCT
GTAA

(5) Codon-optimized *Se*Fdx-5 (GenBank Accession No. SYNPCC7942_0814)

ATGGCCCATACCATTGTGACCAACACCTGCGAAGGTGTGGCAGATTG
CGTGGATGCCTGCCCGGTGGCCTGCATTCAGGAAGGTCCGGGTCGCAA
TCAGAAAGGCACCACCTGGTACTGGATCGATTTCAGCACCTGCATCGATT
GCGGCATTTGCCTGCAGGTGTGCCCGGTGGAAGGTGCCATTCTGCCGG

AAGAACGCCCGGAACTGCAGCAGACCCCGTAA

(6) Codon-optimized *Se*Fdx-6 (GenBank Accession No. SYNPCC7942_0698)

ATGCCGAGCATTCGCTTCATTCGCGAGGATAAAGAAGTTTTTGCCGC
CGATGGCGCAAACCTGCGCTTTAAAGCCGTGGAAAACCAGGTGGACCTG
TATACCTTCGGCGGCAAGATGATGAACTGCGGCGGTTATGGTCAGTGCG
GTACCTGCATTGTGGAGATTGTGCAGGGCGCCGAAAATCTGAGCCCGCG
CACCAGCTTCGAAGAACGCAAGCTGAAACGTAAGCCGGATAGCTATCGC
CTGGCCTGTCAGGCCACCGTGAATGGTCCGGTGACCGTTCTGACCAAAC
CGAACCCGAAAGAAGCCCAGCGCGAAACCCTGATTGCACAGGATCTGGC
CCGCCCGATTCCGGTTACCGCACCCCCTGCCCTGCCGCAGACCGAAAC
CGAGGTGGCAGGTGATCCGCCGAGCATTGCCACCGCCGAAACCTAA

(7) Codon-optimized *Se*Fdx-7 (GenBank Accession No. SYNPCC7942_0338)

ATGGCCACCTATCAGGTGGAAGTGATTTACCAGGGCCAGAGCCAGA
CCTTTACCGCCGATAGCGATCAGAGCGTGCTGGATAGCGCACAGGCCGC
AGGTGTTGATCTGCCGGCCAGCTGCCTGACCGGTGTGTGCACCACCTGT
GCCGCACGCATTCTGAGCGGCGAAGTGGATCAGCCGGATGCCATGGGT
GTGGGTCCGGAACCGGCCAAACAGGGTTATACCCTGCTGTGCGTGGCAT
ATCCGCGCAGCGACCTGAAAATCGAGACCCACAAAGAAGATGAGCTGTA
TGCCCTGCAGTTTGGTCAGCCGGGTTAA

(8) *Cg*Fdx-1 (GenBank Accession No. NCgl1057)

ATGACATACACAATCGCACAGCCCTGCGTTGACGTCTTGGATCGTGC
CTGCGTTGAAGAATGCCCAGTAGATTGCATCTACGAAGGTAAGCGCATGC
TGTACATCCACCCGGATGAGTGCGTTGACTGTGGTGCATGTGAGCCTGC
TTGCCCAGTTGAGGCAATCTTCTACGAGGACGATGTCCCAGACGAATGG
CTTGACTACAACGATGCCAACGCTGCATTCTTCGATGATCTGGGCTCCCC
AGGTGGTGCGGCTAAGCTTGGACCACAAGATTTTGATCACCCAATGATCG
CTGCGCTGCCGCCTCAGGCATAA

(9) *Cg*Fdx-2 (GenBank Accession No. NCgl0526)

TCACACTTGCGTTTCTGGCGTGGTCAACGAAAGATCCATGCCTTCGG
TGACCTTGATTTGGCAAGACAAACGGGAGCAATCCTCACGGTCCACGGC
AGCACCCCACAGCATTTCATCTTCCATCTCCTCCATTGGGGGAAGCGCAT
CATACTGTGCAGGGTCAACAAACACATGGCAGGTTGCACACGATAAGGAA
CCGCCGCATTCAGCAACAATTCCAGGCACTCCGTTTCGGACTGCGGTCT
CCATTACTGAATCACCAACAGTCGCCTCGATGGTGCGGGTTTTGCCAGCA
TGATCAATGAAATGAATAGTAGACAT

(10) *Cg*Fdx-3 (GenBank Accession No. NCgl2856)

CTAGTTCTGGTTCTGTGGCGGCAGCACCGCGACGAGCTGGGCGTC
GAAGTCCTGCGGACCCAGGCTGGCGGCACCGCCTGGCGAACCGAGGT
CGTCGAAAAAGGCGGCGTTAGCGCCGGTGTAGTCCCACCATTCGTGGG
GAACATCATCTTCGTAGAAGATGGCTTCAACCGGGCAGACGGGCTCGCA
GGCACCGCAGTCGACGCACTCATCGGGGTGGATGTAGAGCATCCGTTTG
CCCTCGTAGATGCAGTCCACGGGACATTCCTCGACGCAGGCTCGATCCA
GGACATCAACGCAGGGCTGGGCGATTGTGTAGGTCAT

2. Ferredoxin reductases

(1) *Cg*FdR-1 (GenBank Accession No. NCgl2719)

ATGACAACTCCCCTGCGCGTAGCCGTCATCGGAGCTGGCCCTGCTG
GCATTTACGCATCCGACCTCCTCATCCGCAATGAAGAGCGCGAAGTGTTC
GTTGACCTTTTCGAGCAAATGCCTGCACCGTTCGGACTCATCCGTTACGG
CGTTGCTCCAGACCACCCACGCATCAAGGGCATCGTTAAGTCCCTGCAC
AACGTGTTGGACAAGCCACGCCTGCGCCTGCTCGGTAACATTGAAATCG
GCAAAGACATCACCGTCGAAGAACTCCGCGACTACTACGATGCAGTCGT
GTTCTCCACCGGCGCAGTTGCAGACCGCGACCTCAACATCCCCGGAATT
GAAGCAGAAGGCTCCTTCGGTGCCGGCGAGTTCGTTGGCTTCTACGACG
GCAACCCACGCTTCGAGCGCTCCTGGGATCTGTCTGCACAGTCCGTCGC
TGTTATCGGCGTTGGTAACGTCGGCCTCGACGTAGCCCGCATCCTGGCT
AAGACAGGCGACGAGCTCAAAGTCACCGAAATTTCCGACAACGTCTACG
ACTCCCTCAAAGAAAACAAGGCCACTGAAGTGCACGTTTTCGGACGTCG
TGGCCCAGCACAGGTCAAGTTCACCCCACAGGAACTCAAAGAACTCGAC
CACTCCCCCACCATCAACGTGGTTGTTGATCCAGAAGACATCGACTACGA
CGGCGCCTCTGAAGAAGCCCGCCGCGCATCCAAGTCCCAGGACCTGGT
CTGCCAGATCCTGGAACAGTACGCAATCCGCGAGCCAAAGGACGCTCCG
CACACCCTGCAGATCCACCTCTTTGAAAACCCAGTTGAGGTTCTTCAAAA
GGACGGCAAGGTTGTTGGCCTGCGCACCGAACGCACCTCACTTGATGG
CAACGGCGGCGTAAACGGAACCGGCGAATTCAAGGACTGGCCAGTCCA
GGCTGTCTACCGCGCAGTCGGCTACAAGTCCGACCCCATCGACGGCGT
CCCATTCGATGAGAACAAGCACGTCATCCCTAATGACGGCGGACATGTCC
TCACCGCTCCAGGCGCAGAACCAGTACCAGGCCTCTATGCAACCGGCTG
GATCAAGCGTGGACCAATCGGTCTAATCGGCAACACCAAGTCCGACGCC
AAGGAAACCACCGACATCCTCATCAAGGATGCCGTCGCCGGTGTACTTG
AAGCTCCAAAGCACCAGGGCGAAGAAGCCATCATCGAGCTTCTCGATTC
CCGCAACATCCCATTCACCACCTGGGAAGGCTGGTACAAACTCGACGCA
GCAGAGCGCGCACTCGGTGAAGCCGAAGGCCGCGAGCGCAAGAAGATT
GTTGATTGGGAAGAAATGGTCCGCCAGGCCCGCGAAGCTCCAGCAATTG
TCTAA

(2) *Cg*FdR-2 (GenBank Accession No. NCgl2658)

ATGTCTCGCCCTTTGCGTGTTGCCGTTGTCGGTGCAGGTCCAGCAG
GAATCTACGCGTCTGATTTGTTGATGAAATCCGACACGGACGTGCAGATT
GATCTTTTTGAACGTATGCCAGCGCCTTTCGGTTTGATCCGTTATGGTGTT
GCGCCTGATCACCCTCGCATCAAGGGCATCGTGAAGTCCCTGCACAATG
TGATGGACAAGGAGCAGCTGCGTTTCTTGGGCAACATTGAGGTCGGCAA
GGACATCACTGTTGAGGAGTTGCGTGAGTTTTATGACGCGATCGTGTTCT
CCACTGGCGCTACTGGCGACCAGGATCTTCGGGTTCCAGGTTCTGATCT
GGAAGGTTCGTGGGGCGCTGGCGAGTTCGTTGGTTTCTATGATGGCAAC
CCGAACTTTGAACGCAACTGGGATCTTTCTGCTGAGAAGGTAGCGGTTG
TTGGTGTCGGTAACGTGGCGTTGGACGTTGCTCGTATTTTGGCGAAGAC
TGGCGATGAGCTGCTAGTTACTGAAATCCCTGACAATGTCTATGAGAGCT
TGGCTAAGAATCAGGCTAAGGAAGTGCACGTTTTTGGTCGTCGTGGACC
TGCTCAGGCGAAGTTCACTCCGTTGGAGCTGAAGGAACTTGACCATTCC

GACACCATCGAGGTGATCGTGAACCCTGAGGACATTGATTACGATGCAGC
TTCGGAGCAGGCTCGTCGTGATTCCAAGTCTCAGGACCTCGTGTGCCAG
ACTTTGGAAAGCTACGCGATGCGCGATCCTAAGGGCGCTCCTCACAAGC
TGTTCATTCACTTCTTTGAGTCCCCAGTGGAGATCCTCGGTGAGGACGG
CAAGGTTGTTGGCCTCAAGACTGAGCGTACTCAGCTGGACGGCAACGGT
GGCGTGACTGGCACCGGCGAGTTCAAGACCTGGGATATGCAGTCAGTTT
ACCGCGCGGTAGGTTACCGTTCTGATGCGATCGAGGGTGTTCCTTTTGA
CGATGAGCGCGCGGTTGTCCCCAACGACGGCGGCCACATCATCGATCCT
GAGGTCGGCTCCCCCATCACTGGCCTGTACGCCACTGGCTGGATCAAGC
GTGGCCCAATTGGACTGATCGGCAACACCAAGTCCGACGCCAAGGAAAC
CACTGAGATGCTGCTTGCTGATCACGCTGCTGGTTCTTTGCCTGCGCCT
GCAAAGCCTGAGTTGGAGTCCATCATTGAGTTCCTCGATGAGCGCAAGG
TTGCGTTCACCACATGGGATGGCTGGCACCTGCTGGATGCTGCGGAGCG
CGCGCTGGGTGAGCCTGAGGGCCGCGAGCGCAAGAAGATCGTTGAGTG
GAATGACATGGTGCGCCATGCTCGTCCAGAATACGACATCTAA

(3) *Se*FdR-1 (GenBank Accession No. SYNPCC7942_0978)

ATGTTGAATGCGAGTGTGGCTGGCGGAGCAGCTACCACCACCTATG
GCAACCGGCTCTTTATCTATGAAGTGATCGGTCTGCGCCAAGCCGAGGG
CGAACCGTCCGACAGCTCAATCCGCCGTAGTGGCAGCACCTTCTTCAAG
GTGCCTTACAGCCGGATGAATCAAGAAATGCAACGGATTTTGCGCCTTGG
CGGCAAAATCGTTAGCATCCGGCCTGCGGAGGAAGCAGCCGCGAATAAT
GGTGCGGCTCCTCTACAGGCAGCAGCTGAAGAACCTGCTGCAGCACCA
ACCCCCGCTCCGGCTGCCAAAAAACATTCAGCCGAAGACGTGCCTGTCA
ATATCTACCGGCCTAACAAGCCTTTCGTAGGCAAGGTGCTCTCGAACGAG
CCCTTGGTTCAAGAAGGCGGGATTGGTGTTGTGCAGCACCTCACCTTCG
ATATTTCGGAAGGCGATCTGCGCTACATCGAAGGTCAAAGTATCGGGATTA
TCCCGGATGGCACCGATGACAAAGGCAAGCCGCACAAGCTCCGTCTTTA
CTCGATCGCATCCACTCGCCACGGCGACCACGTGGATGACAAAACCGTC
TCGCTGTGCGTGCGCCAGCTGCAGTACCAGAACGAAGCCGGCGAAACG
ATTAATGGCGTCTGCTCGACTTTCCTCTGTGGTCTGAAGCCAGGCGATGA
CGTCAAGATCACCGGTCCTGTGGGCAAAGAAATGCTCCTACCGGCGGAC
ACAGACGCCAACGTGATCATGATGGGTACTGGCACCGGGATTGCTCCGT
TCCGAGCCTACCTATGGCGGATGTTTAAAGACAACGAGCGAGCCATCAAC
AGCGAGTATCAATTCAACGGCAAGGCTTGGTTGATCTTCGGGATTCCGAC
GACCGCCAACATCCTCTACAAAGAGGAGCTGGAAGCGCTGCAGGCTCAG
TATCCAGATAACTTCCGCCTGACCTACGCGATCAGCCGCGAGCAGAAAAA
TGAAGCGGGCGGCCGGATGTACATCCAAGACCGCGTCGCTGAACATGCT
GACGAGATCTGGAACCTACTCAAGGACGAAAAAACCCACGTCTATATCTG
TGGTTTGCGTGGCATGGAAGATGGGATCGATCAAGCCATGACCGTCGCA
GCTGCCAAGGAAGATGTGGTTTGGTCTGACTACCAACGCACCCTCAAGA
AAGCGGGTCGTTGGCATGTTGAAACCTACTAG
